# Short knee radiographs can be inadequate for estimating TKA alignment in knees with bowing

**DOI:** 10.1186/s43019-019-0020-4

**Published:** 2020-01-01

**Authors:** Sung-Mok Oh, Seong-Il Bin, Jae-Young Kim, Bum-Sik Lee, Jong-Min Kim

**Affiliations:** 1 Nanoori Hospital, 156, Jange-ro 156, Bupyung-gu, Incheon, 21353 Republic of Korea; 2 Department of Orthopedic Surgery, Asan Medical Center, University of Ulsan College of Medicine, 88, Olympic-ro 43-gil, Songpa-gu, Seoul, 05505 Republic of Korea

**Keywords:** Knee, Arthroplasty, Alignment, Bowing, Short radiograph, Full-length radiograph

## Abstract

**Purpose:**

The aim of this study was to compare the discrepancy of alignment categorization in total knee arthroplasty (TKA) between the anatomical femorotibial angle (aFTA) measured on short knee radiographs and the mechanical hip–knee–ankle axis angle (mHKA) measured on full-length radiographs in knees with and without bowing.

**Methods:**

From January 2014 to June 2017, 107 of 526 osteoarthritic knees at our hospital were found to have femoral or tibial bowing. Bowing was defined as a femoral bowing angle (FBA) > 3° or < − 3° or a tibial bowing angle (TBA) > 2° or < − 2° in full-length preoperative radiographs. Among 419 knees without bowing, we selected 107 knees as a control group using propensity-score matching. Postoperative alignments were categorized by aFTA in short knee radiographs and mHKA in full-length radiographs into neutral (2° ≤ aFTA≤7°, − 3° ≤ mHKA≤3°), varus (aFTA< 2°, mHKA<− 3°), and valgus (aFTA> 7°, mHKA> 3°) alignments. We compared the categorization of alignments between knees with and without bowing using the McNemar test and used logistic regression to find factors for the alignment discordance.

**Results:**

Coronal alignment was discordant in 26.2% of the knees with bowing and 13.1% of the knees without bowing (*p* < 0.001). FBAs were a significant factor affecting the discordance of alignment categorization (OR = 1.152, 95%CI 1.038–1.279, *p* = 0.008).

**Conclusion:**

Short knee radiographs are insufficient for estimating coronal alignment after TKA, particularly in knees with femoral bowing.

**Level of evidence: III:**

Retrospective comparative study.

## Introduction

Postoperative coronal alignment is an important factor in determining the longevity of a total knee arthroplasty (TKA) procedure [1–8]. The mechanical hip–knee–ankle axis angle (mHKA) measured from full-length radiographs is the gold standard for estimating coronal alignment [9]. However, this measure requires special equipment, generates extra costs, and irradiates the pelvic organs [10]. For these reasons, the anatomical femorotibial angle (aFTA) measured from short knee radiographs is often used in clinical studies to estimate the alignment of TKAs [1, 6, 7]. The aFTA is used in many knee-scoring systems, including the modified Hospital for Special Surgery (HSS) knee score [11], the Knee Society Score (KSS), and even the revised KSS [12].

Interestingly, studies that have used short knee radiographs and those that utilized full-length radiographs to determine the longevity of TKAs in accordance with the postoperative alignment have reported different results. The former have reported that a neutral alignment produces a superior longevity than outliers [1, 6, 7]. The latter studies have indicated no differences between neutral alignment and outliers in terms of TKA longevity [2, 3, 8, 13]. Notably however, although many studies of TKAs have been performed using short knee radiographs to evaluate postoperative alignment [1, 6, 7, 14], the question of whether short knee radiographs can replace full-length radiographs remains disputed. Some previous reports have found a small difference of less than 1° between the aFTA on short knee radiographs and mHKA on full-length radiographs and a high correlation (*r* = 0.8) [15, 16]. By contrast, other investigations have reported a large difference of up to 7.2° and a low correlation (*r* = 0.34–0.41) between the aFTA and mHKA [10, 17–19]. The reported alignment categories of neutral, varus, and valgus for the aFTA and mHKA are discordant over a variable range (15–70%) [17, 20–22].

Extra-articular deformities, such as femoral and tibial bowing, could have caused such discrepancies in the results of previous studies [17, 20–22]. To date however, no studies have reported on the percentage of neutral alignments of the aFTA measured from short knee radiographs that would also be neutral alignments of the mHKA on a full-length radiograph in knees with or without bowing. Hence, we performed a direct comparison of the discordance in alignment categorization between knees with and without bowing in our present study. We speculated that these analyses would provide meaningful data that indicated why previous studies showed different results.

We hypothesized that coronal bowing itself could cause aFTA categorizations measured from short radiographs to be less accurate than the corresponding mHKA categorizations measured from full-length radiographs in patients who have undergone TKA. We thus investigated the prevalence of discordance between these two sets of categorizations in accordance with the existence of femoral or tibial bowing.

## Materials and methods

**Fig. 1:**
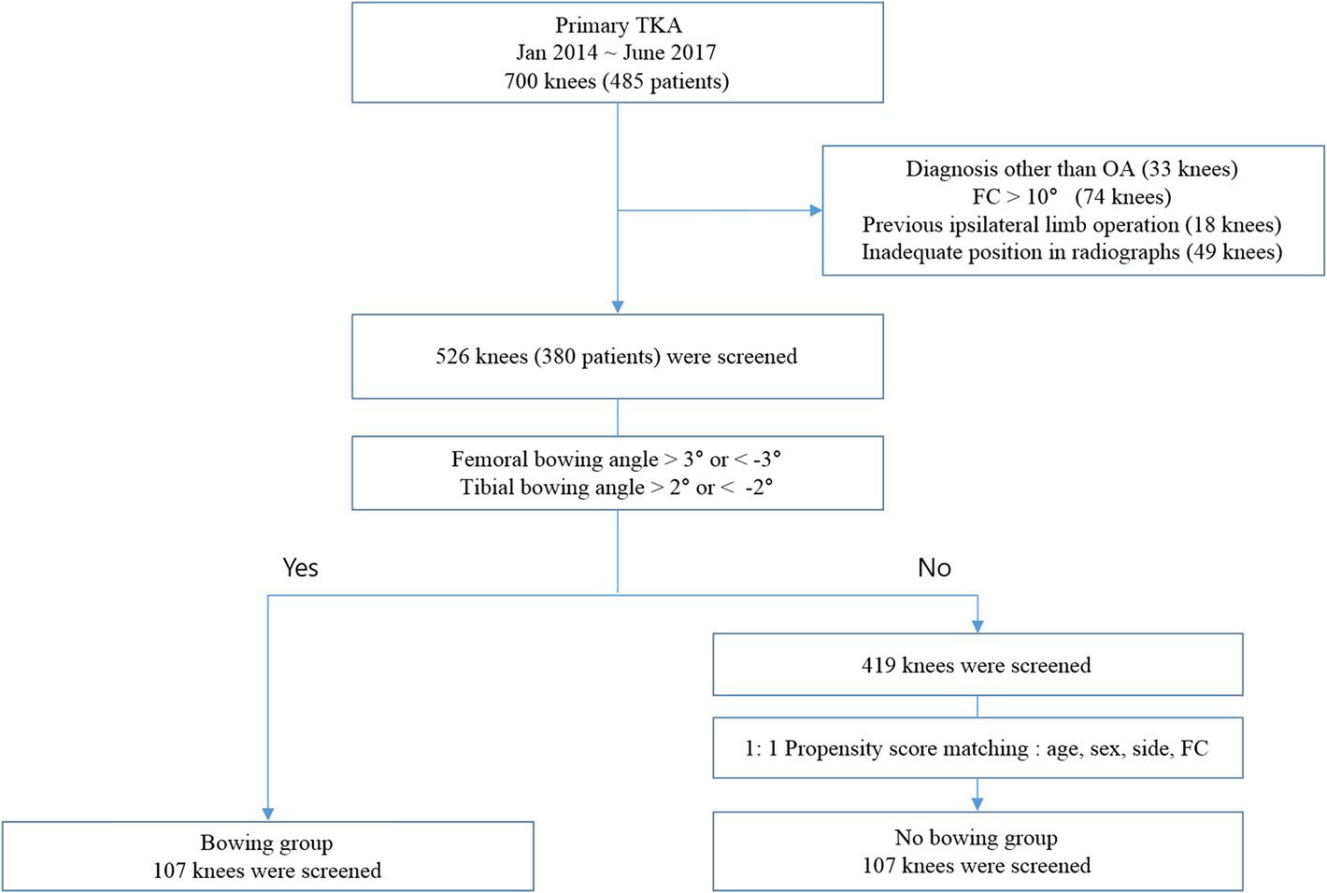
Study flow diagram. TKA, total knee arthroplasty; OA, osteoarthritis; FC, flexion contracture

Preoperative and postoperative radiographs of 485 patients (bilateral 215, unilateral 270) who underwent a TKA at our hospital between January 2014 and June 2017 at our hospital were retrospectively reviewed. Among the 700 knees of these 485 consecutive cases, we applied the following exclusion criteria: diagnosis other than primary osteoarthritis (e.g., rheumatoid arthritis, osteonecrosis, or traumatic osteoarthritis), flexion contracture of over 10°, previous history of operation on the ipsilateral limb (e.g., hip arthroplasty, internal fixation of a femoral or tibial fracture, unicompartmental knee arthroplasty, high tibial osteotomy), or inadequate rotation on radiographs. In all, 526 knees of 380 patients were screened in our current study. Among these 526 knees, 107 were identified with femoral or tibial coronal bowing in preoperative weight-bearing full-length radiographs. We matched 107 knees without bowing using age, sex, side, and preoperative flexion contracture in accordance with the propensity score using R software. A total of 214 knees of 182 patients (107 each in the bowing and no bowing groups) were therefore enrolled in the final study cohort (Fig. 1).

### Preoperative radiographs

**Fig. 2:**
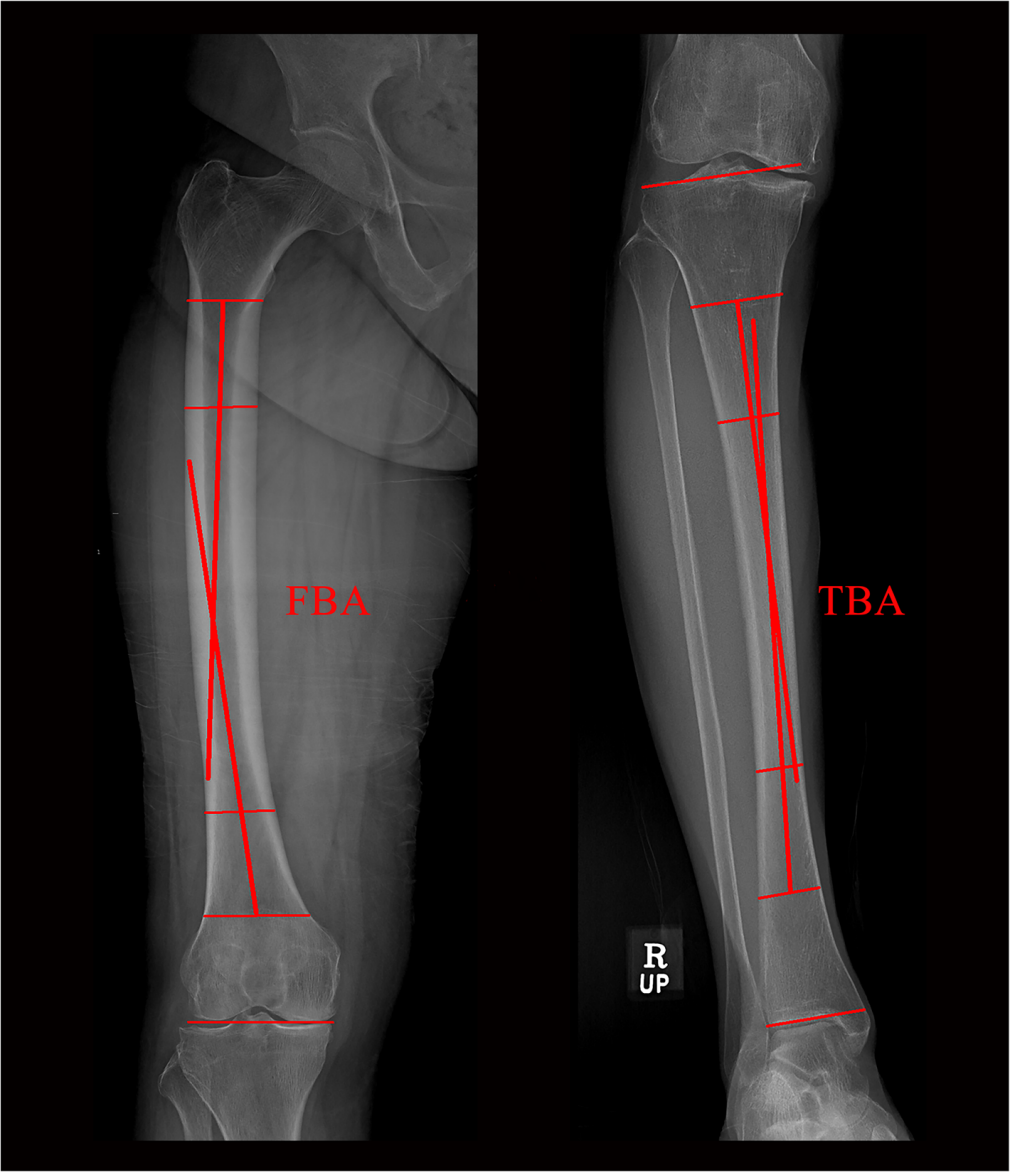
Preoperative full-length (14 × 51 in. grid cassette) radiograph showing the measurement of the femoral bowing angle (FBA) and tibial bowing angle (TBA). The FBA was defined as the angle between the line connecting the points bisecting the femur at 0 cm and 5 cm below the lowest portion of the lesser trochanter and the line connecting the points bisecting the femur at 5 cm and 10 cm above the lowest portion of the lateral femoral condyle [23]. The TBA was defined as the angle between the line connecting the points bisecting the tibia at 5 cm and 10 cm from the highest portion of the lateral tibial plateau and the line connecting the points bisecting the tibia at 5 cm and 10 cm from the lowest portion of the tibial plafond [19].

The femoral bowing angle (FBA) and tibial bowing angle (TBA) were determined from preoperative standardized full-length hip–knee–ankle weight-bearing anteroposterior (AP) radiographs, measuring 14 × 51 in.. Full-length weight-bearing radiographs were obtained with the subjects standing barefoot in a position in which the patella was oriented forward (Fig. 2). Femoral bowing was defined as an FBA > 3° or < − 3° [23]. Tibial bowing was defined as a TBA > 2° or < − 2° [19, 24]. All lateral bowing was recorded as positive, whereas medial bowing was recorded as negative.

### Postoperative radiographs

**Fig. 3:**
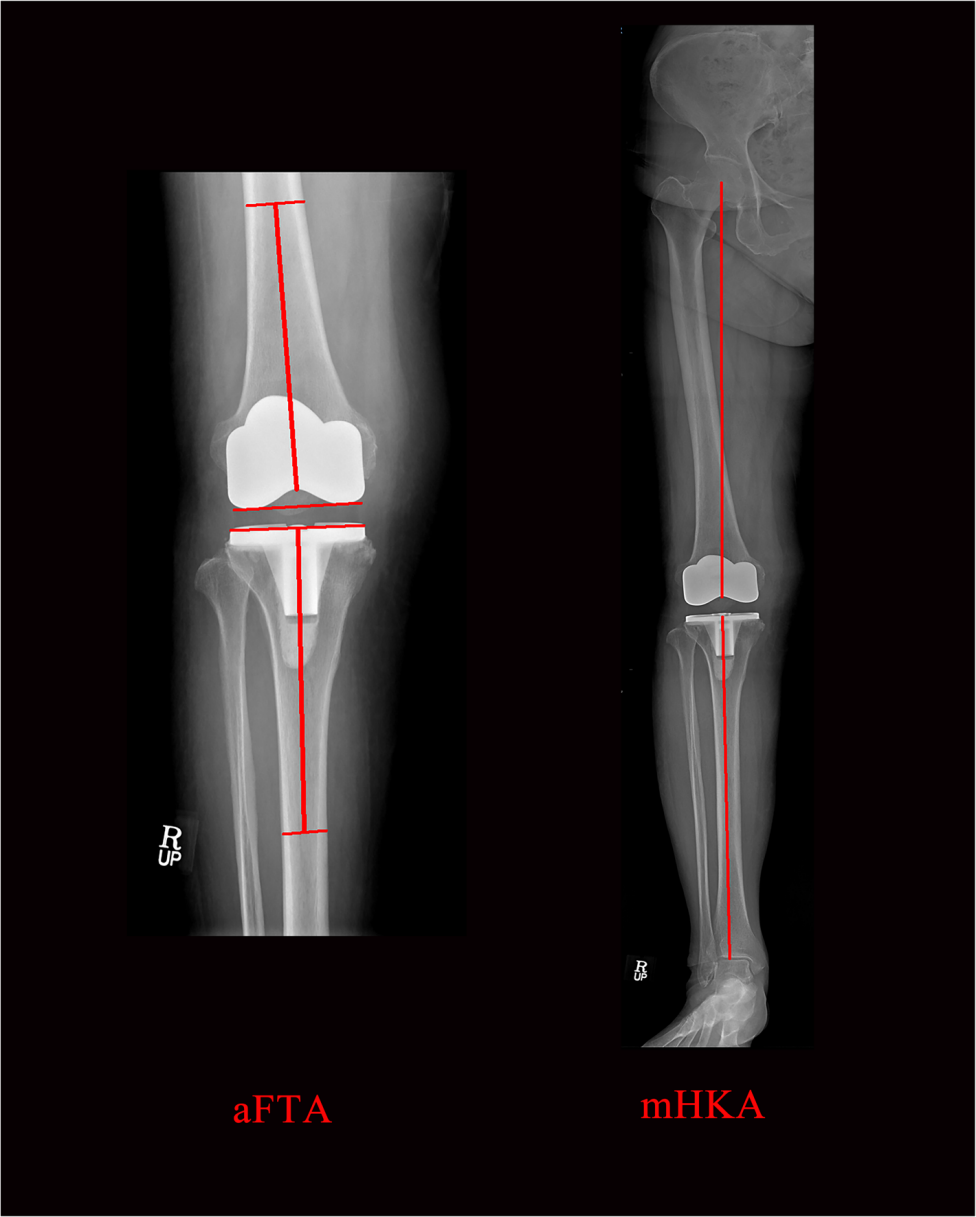
Postoperative short (14 × 17 in. grid cassette) and full-length (14 × 51 in. grid cassette) radiographs showing the measurement of anatomical femorotibial angle (aFTA) and mechanical hip–knee–ankle axis angle (mHKA) The postoperative aFTA was measured using two lines, the first aligned from the bisection point of the femur, 15 cm above the lowermost portion of the femoral component to the deepest part of the center of the femoral component, and the second from the bisecting point of tibia 15 cm down from the uppermost portion of the tibial component [19]. Postoperative mHKA was made between the two lines, the first aligned from the center of the femoral head to the deepest part of the center of the femoral component, and the second from the midpoint of the inner extension of the tibio-talar joint to the center of the tibial component [14].

The aFTA was measured from routine 14 × 17 in. weight-bearing AP radiographs, with the patellae facing forward and the legs in full extension, and the mHKA was measured from routine full-length hip–knee–ankle weight-bearing AP radiographs measuring 14 × 51 in. (Fig. 3). Routine weight-bearing AP and lateral knee radiographs (14 × 17 in. cassette) and full-length standing films (14 × 51 in. cassette) were taken at 6 weeks, 3 months, 6 months, 1 year, and every 2–3 years thereafter. Pairs of standing short AP knee radiographs and standing full-length AP radiographs, taken on the same day and in the same position, were evaluated. We measured parameters from radiographs taken within 1.5 years. The alignments on short knee radiographs were classified as neutral (2° ≤ aFTA ≤7°), varus (aFTA < 2°) or valgus (aFTA > 7°) [1, 7, 20, 25]. Postoperative mHKA was expressed as a deviation from 0°, with a positive value for the valgus direction and negative value for the varus direction. The alignments determined from full-length radiographs were also classified as neutral (− 3° ≤ mHKA ≤3°), varus (mHKA < − 3°), or valgus (mHKA > 3°) [2, 8, 13] .

To evaluate intra- and inter-observer reliability, 20 knees were randomly selected, and all parameters were measured twice by two observers with a 2-week interval between measurements. The reliability of the data was expressed in terms of the intraclass correlation coefficient (ICC). The ICC values were over 0.90 in all cases and for all radiographic parameters except for the TBA. Those for the TBA were also greater than 0.85 however. As the reproducibility of all measurements was good to excellent, the parameters measured by only one experienced orthopedic surgeon were used in this study. The measurements were made using the tools from a picture-archiving communication system (PACS). The least detectable angular change was 1°.

### Statistical analysis

The parameters of the study groups, including FBA, TBA, aFTA, and mHKA, are given as means and standard deviations (SDs) (Table 1). Discordance between the aFTA and mHKA with regard to the existence of bowing was investigated using the McNemar test. Comparisons of the differences between the aFTA and mHKA according to knee bowing were analyzed using the Mann–Whitney U test. The association between bowing and discordance between the categorization of aFTA and mHKA alignment was investigated using univariate logistic regression analyses. The results are presented as odds ratios (OR) and 95% CIs. All of the statistical analyses other than propensity score matching were carried out using SPSS for Windows version 21.0 (SPSS, Chicago, IL). *P* values of < 0.05 were considered significant.

**Table 1:** Demographic and radiological data

	No bowing	Bowing	*p* value
	(*n* = 107)	(*n* = 107)	
Age (years)	70.01 ± 5.37	70.10 ± 5.81	0.968^a^
Sex	M 3 F 104	M 5 F 102	0.471^b^
Side	R 55 L 52	R 58 L 49	0.681^b^
FC (°)	5.66 ± 3.46	5.79 ± 3.71	0.697^a^
Bowing type	none	Femur only (*n* = 64) Tibia only (*n* = 24) Femur & Tibia (*n* = 19)	
|FBA| (°)	1.08 ± 1.02	4.65 ± 2.33	< 0.001^a^
Femoral bowing direction	none	M 14@L 69	
|TBA| (°)	0.77 ± 0.78	1.91 ± 1.60	< 0.001^a^
Tibial bowing direction	none	M 25@L 18	
aFTA (°)	3.91 ± 2.08	5.11 ± 2.58	< 0.001^a^
mHKA (°)	−0.83 ± 1.84	−1.04 ± 2.27	0.379^a^
aFTA-mHKA difference (°)	4.74 ± 1.87	6.14 ± 2.49	< 0.001^a^

*FC* Flexion contracture, *|FBA|* absolute value of femoral bowing angle, *|TBA*| absolute value of tibial bowing angle, *M* medial, *L* lateral, *aFTA* anatomical femorotibial angle, *mHKA* mechanical hip–knee–ankle axis angle

^a^Mann–Whitney U test

^b^Fisher’s exact test

## Results

Knees with femoral bowing made up 15.7% (83/526) of the total in the initial patient population whereas 8.2% (43/526) of knees had tibial bowing in this initial cohort. Nineteen (3.6%) of 526 knees had both femoral and tibial bowing. The mean angle differences between the aFTA and mHKA were 4.74° ± 1.87° (SD) for the no bowing group and 6.14° ± 2.49° (SD) for bowing group which was a significant difference (*p* < 0.001; Table 1).

In the no bowing study group, 86.0% (92/107) of the TKAs showed a neutral alignment based on the aFTA on short knee radiographs. However, 3.3% (3/92) of these TKAs that appeared to have a neutral alignment from the aFTA on short knee radiographs were found to have varus or valgus alignments based on the mHKA on full-length radiographs. In the categorization of alignments as neutral, varus, and valgus, 13.1% (14/107) of our study subjects with no bowing were found to be discordant. In the bowing group, 76.6% (82/107) of the TKAs showed a neutral alignment from the aFTA determination on short knee radiographs. However, 14.6% (12/82) of these TKAs that appeared to have a neutral alignment from the aFTA measurements on short knee radiographs were found to have varus or valgus alignments based on the mHKA on full-length radiographs. With regard to the categorization of neutral, varus, or valgus alignments, 26.2% (28/107) of our study subjects with knee bowing were discordant (Table 2). When comparing the no bowing and bowing groups, the discordance ratio was significantly different (13.1% in the no bowing group vs. 26.2% in the bowing group, *p* < 0.001; Table 3). The discordance among alignment categorizations was not affected by the degree of TBA (*p* = 0.704, OR 1.035, 95% CI 0.867–1.235), but affected by the degree of FBA (*p* = 0.008, OR 1.152, 95% CI 1.038–1.279). For each 1° increase in the FBA, the odds of discordance were 1.152 times greater. When the FBA increased more than 5°, the odds of discordance was almost doubled.

**Table 2:** Agreement in the categorization of lower-limb deformity according to the existence of bowing

		aFTA in short-leg AP-measured deformity			Total
		Varus	Neutral	Valgus	
No bowing group					
mHKA on Full-Length AP measured deformity	Varus	4	2	0	6
	Neutral	6	89	5	100
	Valgus	0	1	0	1
	Total	10	92	5	107
Bowing group					
mHKA in full-length AP measured deformity	Varus	6	10	1	17
	Neutral	3	70	12	85
	Valgus	0	2	3	5
	Total	9	82	16	107

**Table 3:** Lower-limb deformities in accordance with the existence of bowing

	Accordance	Discordance	Total
No bowing group	93 (86.9%)	14 (13.1%)	107 (100%)
Bowing group	79 (73.8%)	28 (26.2%)	107 (100%)

McNemar test, *p* < 0.001

## Discussion

To the best of our knowledge, our current study is the first to suggest discordance between the alignments in knees with or without bowing that are categorized by the aFTA from short knee radiographs and the mHKA on full-length radiographs. In knees with bowing, alignment categorizations were discordant at a higher rate (26.2%) than in knees without bowing (13.1%) (*p* < 0.001). Bowing that could not be accessed using short knee radiographs has been suggested previously as the cause of discordance between the aFTA and mHKA [19, 21]. Notably, the incidence of bowing is especially high in Asian patients [24, 26, 27]. If the proportion of bowing is higher in a given patient cohort, there would be a greater risk of overestimating or underestimating the alignment. The direct comparisons of the alignment discordance between knees with and without bowing would help to more clearly elucidate the effects of bowing. The results of our present study strongly support that full-length radiographs should be used to evaluate postoperative TKA alignment in knees with bowing.

Alignment discordance between short knee radiographs and full-length radiographs has been reported at rates ranging from 15% to 70% [17, 20–22]. However, previous studies have not considered discordance according to the existence of bowing. Park et al. [20] reported that 33% of their subjects had discordant alignment in the aFTA and mHKA on postoperative images (neutral criteria: 2.4° ≤ aFTA ≤7.2°, mHKA ≤ ±3°). Abu-Rajab et al. [22] reported that only 30% of categorizations were done correctly with the aFTA (neutral criteria: aFTA < 2°, mHKA 0°). Van Raaji et al. [17] reported that 15% of varus alignment knees determined by mHKA were classified as having no varus deformity when categorized by aFTA on preoperative radiographs (varus criteria: aFTA < − 2°, mHKA < 0°). Lee et al. [21] recorded only a 73% accuracy between aFTA and mHKA categorization (neutral criteria: 3° ≤ aFTA ≤9°, mHKA ≤ ±3°). Although there have been variable definitions of neutral alignment [17, 21, 22], we here confirmed that the discordance of alignment categorization between the aFTA and mHKA in knees without bowing (13.1%) was lower than the results of previous studies and in the knees with bowing in our present cohort (26.2%) [17, 20–22].

Our present analyses also demonstrated that the categorization discordance was greater for knees with a greater FBA (OR 1.152, 95% CI 1.038–1.279, *p* = 0.008). It has been indicated previously in one study that knees with a greater FBA (OR = 1.2) and TBA (OR = 1.2) may show a lower accuracy in alignment categorization between aFTA and mHKA [21], and in another report that the FBA and TBA, particularly of the femur, may be major contributors to the divergence between the aFTA and mHKA in TKA patients [19]. Our current findings are compatible with these earlier results in that a greater FBA was found in our present analysis to be a significant factor in the discordance of alignment categorizations. In our current analyses also, 6.0% of the neutral mHKA cases in the no bowing group and 3.5% of the patients with this alignment in the bowing group were categorized by aFTA as having a varus alignment on short knee radiographs. However, 14.1% of those categorized as having a neutral alignment in the mHKA in the bowing group were found to have a valgus alignment in aFTA on short knee radiographs, or nearly triple that of the no bowing group (5.0%). These results also support the conclusion that there is a tendency for knees with bowing to be measured as having a valgus direction when using short knee radiographs. Femoral bowing usually occurs in the lateral direction and tibial bowing is usually found in the medial direction. Lateral femoral bowing could thus make alignments using the aFTA appear more valgus and medial tibial bowing could make alignments with the aFTA appear more varus than the corresponding alignments determined by the mHKA. The wide range of examples of femoral bowing and relatively small angle and small number of instances of tibia bowing could be the reason for these results.

There were several limitations to our study of note. First, the analysis was retrospective and could be biased in terms of patient selection. However, we reviewed full-length preoperative radiographs of 700 consecutive TKA cases over an appreciable time period and matched our subject knees with bowing to those without bowing using a propensity score. Second, our sample cohort comprised mainly female Korean subjects undergoing TKA and our results may not therefore be fully applicable to male patients or Western populations. Third, the definition of femoral and tibial bowing and the method used to measure them may have altered the results. In our current analysis, the definition and measurement methods for femoral bowing and tibial bowing were as described in previous studies [19, 23, 24]. Fourth, the definition of neutral alignment could have affected our findings. The range of neutral alignments using the aFTA is reported to be variable [1, 6, 12, 28]. Ritter and Fang et al. reported in two studies that included 6070 knees with 7.6 years follow-up and 6070 knees with 6.6 years follow-up, respectively, that an aFTA of between 2.5° and 7.4° measured on short knee radiographs should be considered a neutral coronal alignment [1, 7]. In most of the previous studies that have used full-length radiographs, the mHKA was considered neutral if it ranged from − 3° to 3° [2, 4, 8, 13]. Liu et al. reported in their earlier meta-analysis of 10 studies of short knee radiographs and full-length radiographs that an aFTA from 2.5° to 7.4° and mHKA from − 3° to 3° have been regarded as a neutral alignment up to the present time [5]. Park et al. have also reported the discordance of alignment categorization using same criteria for a neutral aFTA and mHKA without consideration of bowing [20]. As the least detectable angular change was 1° in our PACS system, we defined a neutral alignment as 2° ≤ aFTA ≤7° on a short knee radiograph and − 3° ≤ mHKA≤3° on a full-length radiograph. Fifth, as with all radiographic assessments of coronal plane alignments, lower-extremity rotation and flexion contracture could affect the radiological parameters of full-length radiographs [29, 30]. However, we used full-length radiographs that were taken using a detailed uniform protocol and we excluded knees with a preoperative flexion contracture of more than 10°. In addition, there was no patient with genu recurvatum who showed a hyperextension of the knee of more than 10°. Sixth, we only analyzed coronal bowing. However, the results from a previous study on femoral bowing indicated a poor correlation between the parameters in the coronal and sagittal planes [31]. Moreover, in other studies on bowing [19, 23, 24] or reporting on the category of the alignment after TKA [20–22] in the coronal plane, only the parameters measured on films of the coronal plane were used. We speculated that the bowing on the sagittal plane might not significantly affect the coronal alignment of TKAs on radiographs taken in the patella forward position.

## Conclusion

The aFTA measured from short knee radiographs and mHKA measured from full-length radiographs are both used in studies reporting the mid- to long-term outcomes of TKA and in scoring systems to estimate coronal alignment. However, the aFTA measured using short knee radiographs is insufficient for evaluating the coronal alignment after TKA, particularly in knees with femoral bowing. The results of previous studies that have used this approach should therefore be reconsidered, at least in patients with femoral bowing. Full-length radiographs are recommended for analyzing alignment categorizations after TKA.

## Data Availability

The datasets used and/or analyzed during the current study are available from the corresponding author on reasonable request.

## Abbreviations

aFTA: anatomical femorotibial angle
FBA: femoral bowing angle
HSS: Hospital for Special Surgery
KSS: Knee Society Score
mHKA: mechanical hip–knee–ankle axis angle
TBA: tibial bowing angle
TKA: total knee arthroplasty
